# Ideal Gas Thermodynamic Functions For Water

**DOI:** 10.6028/jres.092.004

**Published:** 1987-02-01

**Authors:** Harold W. Woolley

**Affiliations:** National Bureau of Standards, Gaithersburg, MD 20899

**Keywords:** anharmonicities, centrifugal effects, ellipsoidal shell, Hamiltonian, semi-axes, vibrational states

## Abstract

The calculation of ideal gas thermodynamic properties for steam to 10,000 K is examined. Centrifugal effects are included using spectroscopic data for the lowest vibrational levels, with extension to higher bending levels based on estimates from a bending model. Modifications are examined for rotational and vibrational cut-off effects. Uncertainties in obtaining a suitably regularized representation of energy versus bond stretching vibration in approaching the dissociation energy region appear relevant to the reliability of the extrapolation.

## Introduction

A calculation made in 1979 of the ideal gas thermodynamic properties for water [1]^1^ is the background for this study on improving the extrapolation to higher temperatures. The procedure under consideration is to calculate the internal partition function and its first two temperature derivatives, using direct summation over vibrational levels.

Direct summation was also used for the 1979 work. It was used not only for vibrational levels, but for rotational ones as well for temperatures up to 230 K. In the earlier work of Friedman and Haar [2], sums over vibrational levels were computed according to a procedure similar to that of Mayer and Mayer [3] for diatomic molecules. Such a formulation is in principle a low temperature form, analogous to a power series in increasing powers of temperature, and as such encounters questions of convergence in extension to very high temperature.

While it is not too difficult in such an approach to include effects of lowest order anharmonicities in their first power contribution, the inclusion of higher power contributions of even the lowest order of anharmonicities including cross product effects and rotational dependence is more complex. The extension to cover still higher order terms such as might satisfactorily describe the approach to the energy of dissociation would involve too many terms of cross product power type for easy confidence in the adequacy of their enumeration. There is in fact no formal end to the number of possible orders of the expansion, and each succeeding order would be enormously more complex than the one before it.

In the presentation that follows, the problem of the myriads of correction terms is circumvented by using direct summation over the three vibrational quantum numbers for this triatomic molecule. The problems which remain are the physical ones of obtaining reliable energies for the vibrational levels and reliable rotational parameters, including the way in which the rotational energy behaves in the extension to high rotational quantum numbers.

1. As a first step, this last item is discussed with a study of an empirical bending model, including its implications as to bending energy.
2. The next topic to be covered is the implied centrifugal thermodynamic effect based on empirical constants fitting the spectroscopically-determined rotational levels as represented by a Watson Hamiltonian [4]. This applies to eight low lying vibrational levels, extending up to 5331 cm^−1^.
3. Following this, the problem of extending the representation of rotational details to high energy and high temperature is considered, including the termination of rotational levels due to lack of bond stability.
4. Finally, considerations are introduced pertaining to a suitable extrapolation for energies of vibrational levels in approaching the dissociation region, a problem that is not regarded as solved.

The calculation range of new estimates for ideal gas thermodynamic functions for water is from 200 K to 10,000 K. It uses

$$Q=\underset{v}{\Sigma }Q_{v}\;\exp\left(-G_{v}hc/kT\right),$$ (1)

with *v* indicating all sets of values for *v*_1_, *v*_2_ and *v*_3_ giving stable vibrational states. *G_v_* is the vibrational energy in cm^−1^ units. *Q_v_* is the rotational partition function for the given vibrational state. This is taken as

$$Q_{v}=Q_{v}^{0}f_{v}+dQ_{v}$$ (2)

with the usual semi-classical relation

$$Q_{v}^{0}=\pi^{1/2}{\left(kT/hc\right)}^{3/2}{\left(A_{v}B_{v}C_{v}\right)}^{-1/2}\sigma^{-1}$$ (3)

where the symmetry number *σ* is equal to 2 for the H_2_0 molecule. *A_v_, B_v_*, and *C_v_* are the principal rotational constants and *dQ_v_* is a “low temperature” quantum correction such as that of Stripp and Kirkwood [5] as used in reference [1]. The centrifugal distortion and stretching effects are here represented by the factor *f_v_*, related to the “Wilson [6] centrifugal effect constant *ρ_v_*” by *f_v_*=exp(*h_v_*), where *h_v_=ρ_v_T.* The treatment of centrifugal effects for eight of the lowest vibrational levels is based on spectroscopic data interpreted with a Watson type rotational Hamiltonian. The extension to high bending quantum number is made on the basis of model estimates.

An examination is also made as to plausible magnitudes for effects of rotational cut-off in the dissociation region. Ad hoc adjustments in the approach to the dissociation energy region have been made to preserve approximate symmetry versus *v*_1_ and *v*_3_ quantum numbers.

For temperatures upward of 200 K, the data and empirical representations of Camy-Peyret et al. [7] could be used for seven vibrational levels above the ground state for which detailed parameters for rotational Hamiltonians are available. These include values for the principal rotational constants *A_v_, B_v_* and *C_v_*, and for *G_v_*, the energy for the vibrational level at zero rotation. Their reported values for *G_v_* were used also for four other vibrational levels. Their *G_v_* data for the (1,1,1) state were not used, as its partner in resonance, (0,3,1), had not apparently been similarly covered. A placement estimate for a (0,4,0) level based on a resonance shift from Benedict [8] was used to complete *G_v_* values for the resonating triad including the (2,0,0) and (1,2,0) levels. For the ground vibrational state, however, the slightly differing results of the more recent analysis by Kyro [9] were accepted in the later calculations. The overall course of vibrational energies versus vibrational quantum number was taken to follow an empirical data fit by Benedict [8], but with some adjustment in the higher energy regions to be consistent with other data, such as dissociation and heats of reaction.

## The Bending Model

On the basis of spectroscopic data it can be inferred that vibrational bending produces large effects on *f_v_* and special effects on the total energy. The lack of extensive data and the impossibility of making reliable long range extrapolations of directly fitted polynomial representations of data have led to the present numerical exploration based on a simple bending model. For this, somewhat crude evaluations of WKBJ integrals have been used, based on an approximate bending potential.

The potential *U* is taken as the product of an empirical basic function *U*_0_ and an empirical correction function *U*_c_, as

$$U=U_{0}\cdot U_{c}.$$ (4)

**Figure 1:**
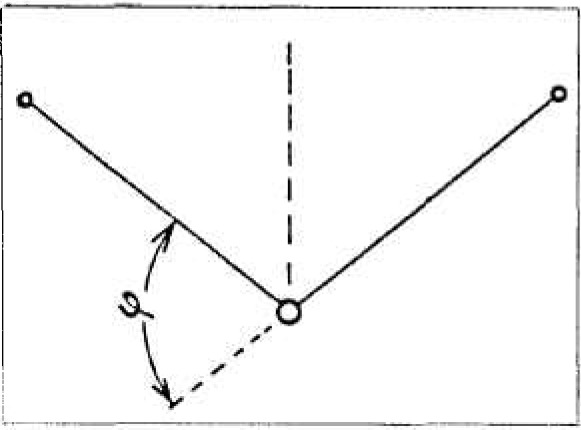
A schematic model of the water molecule.

The basic function *U*_0_ here involves two Lorentz type terms,

$$U_{0}=k_{1}/\left(q_{1}+x\right)+k_{2}/\left(q_{2}+x\right)-k_{1}/q_{1}-k_{2}/q_{2}$$ (5)

with the correction function *U*_c_ taken as

$$\begin{array}{c} U_{c}=\left(1+B_{1}x+B_{2}x^{2}+B_{3}x^{3}-B_{4}gx^{4}\right)\\ ÷\left[1-B_{4}\left(g+1\right)x^{4}\right], \end{array}$$ (6)

where *x*=(*ϕ*^2^ − *ϕ_e_*^2^)/*ϕ_e_*^2^ with *ϕ* as the angle of bending of H-O-H out of a straight line as shown in figure 1. The even power of *ϕ* in the definition of *x* gives symmetry about the angle for full barrier height, at *ϕ*=0. The subscript *e* refers to the equilibrium configuration.

The calculations were made with bonds of fixed length *“d,”* taking the atoms as point masses. The quantum phase integral

$$v_{2}+1/2=m_{H}{\;}^{1/2}d\;h^{-1}\int_{\phi_{\min}}^{\phi_{\max}}{\left[1-\left(1+\cos\;\phi \right)÷\left(m_{O}/m_{H}+2\right)\right]}^{1/2}{\left[G-U\left(i\right)\right]}^{1/2}d\phi$$ (7)

may be put in the form

$$v_{2}+1/2=\left(2^{1/2}/4\pi \right){\left(1+\cos\;\phi_{e}\right)}^{-1/2}\int_{\phi_{\min}}^{\phi_{\max}}\times {\left[1-\left(1+\cos\;\phi \right)/\left(m_{O}/m_{H}+2\right)\right]}^{1/2}\times {\left\{\left[G-U\left(i\right)\right]/B_{e}\right\}}^{1/2}d\phi$$ (8)

for which conventional wave number energy units are convenient. The quantities *m*_O_ and *m*_H_ represent masses of the respective atoms.

In the absence of rotation, *U*(*i*) is identical with *U* of eq (4). For rotation purely about the principal axes there are three cases:

$$\begin{array}{l} \left(i=a\right):U\left(a\right)=U+J_{a}{\;}^{2}A_{e}\left(1-\cos\;\phi_{e}\right)/\left(1-\cos\;\phi \right)\\ \left(i=b\right):U\left(b\right)=U+J_{b}{\;}^{2}B_{e}\left(1+\cos\;\phi_{e}\right)/\left(1+\cos\;\phi \right)\\ \left(i=c\right):U\left(c\right)=U+J_{c}{\;}^{2}C_{e}\left[1+m_{H}{\left(m_{O}+m_{H}\right)}^{-1}\cos\;\phi_{e}\right]/\left[1+m_{H}{\left(m_{O}+m_{H}\right)}^{-1}\cos\;\phi \right]. \end{array}$$ (9)

Evaluations were made using cm^−1^ energy units, with

$$\begin{array}{r} U_{0}=40266.9\left[1/\left(1-0.21507x\right)-1\right]\\ +13464.5\left[1/\left(1+0.6432x\right)-1\right] \end{array}$$ (10)

and

$$U_{c}=\left(1-0.2467x-0.1526x^{2}+0.5678x^{3}+1.3708x^{4}\right)/\left(1+1.7442x^{4}\right).$$ (11)

A value of 75.73 degrees was used for *ϕ_e_*, in adjusting between *A_e_* and *B_e_* indications of a preliminary data fit of Benedict [8].

Energy values on effective potential curves including rotation were approximated by

$$U\left(a\right)=U+20.24J_{a}{\;}^{2}/\left(1.0001-\cos\;\phi \right)$$ (12)

$$U\left(b\right)=U+17.97J_{b}{\;}^{2}/\left(1.0001+\cos\;\phi \right)$$ (13)

and

$$U\left(c\right)=U+9.5J_{c}{\;}^{2}/\left(1+0.0593\cos\;\phi \right)$$ (14)

where 0.0001 has been added in two of the denominators to avoid accidental division by zero.

Values of *v*_2_+1/2 were computed at 17 to 40 suitably spaced values for energy *“E”* or *“G”* up into the 100,000 cm^−1^ region for these three cases of rotation about the principal axes, for several values of each *J_i_*^2^ ranging from zero to 400 in the “*a*” and “*c*” cases and somewhat further in the “*b*” instance. Four point Lagrangian interpolation was then used to obtain energies corresponding to integer *v*_2_ values at each of the chosen *J_i_*^2^ values. Effective values for the principal rotational constants were estimated according to

$$A_{v}=\left(E_{Ja}-E_{0}\right)/J_{a}{\;}^{2}$$ (15)

$$B_{v}=\left(E_{Jb}-E_{0}\right)/J_{b}{\;}^{2}$$ (16)

$$C_{v}=\left(E_{Jc}-E_{0}\right)/J_{c}{\;}^{2}.$$ (17)

**Figure 2:**
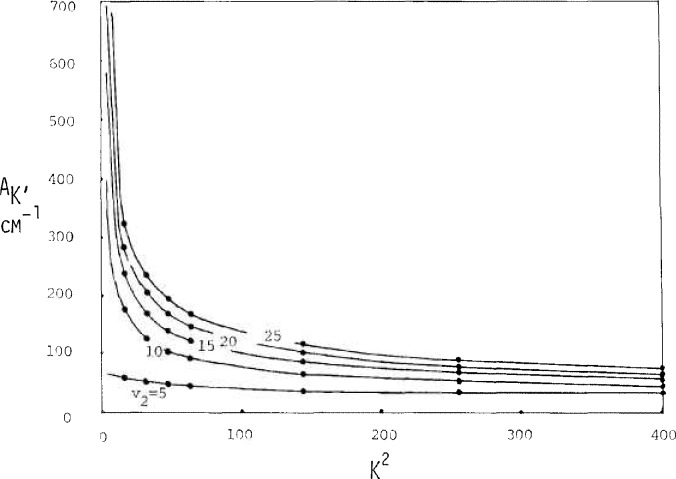
Dependence of the effective rotational constant *A_K_* or *A_Ja_* on *J_a_*^2^ or *K*^2^.

**Figure 3:**
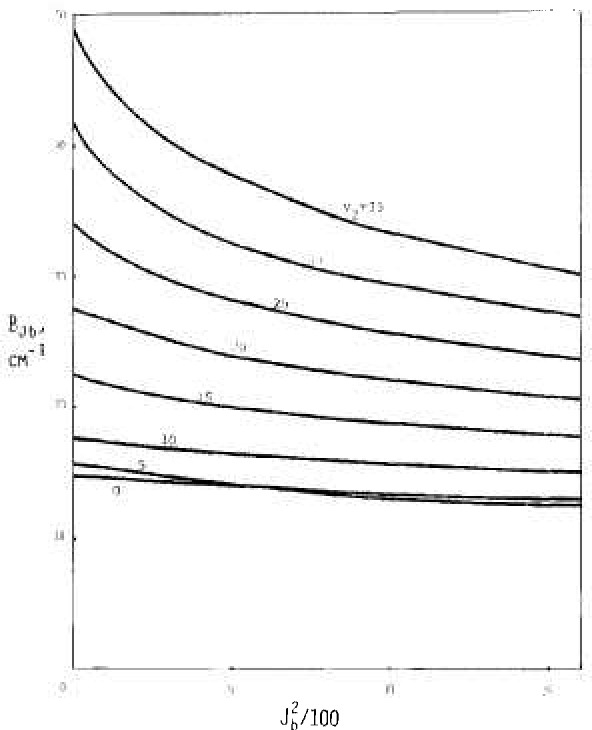
Dependence of the effective rotational constant *B_Jb_* on *A_b_^2^*.

**Figure 4:**
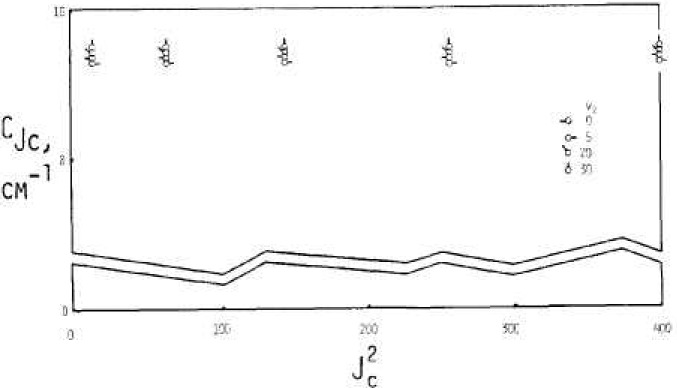
Effective values for the rotational constant *C_Jc_.*

Figures 2, 3, and 4 show results from these calculations. Extrapolation to zero rotation appears reliable for *B_v_* and *C_v_* and for *A_v_* for *v*_2_, small.

Estimates for the centrifugal effect contribution associated with rotation about separate principal axes were obtained in the following way. In the case of the “*B*” rotation, for example, and empirical representation for *B* versus *J_b_*^2^ was used for each of various *v*^2^ values. Numerical quadratures were performed for a partition function contribution as

$${\left(Q\right)}_{B}=\int_{0}^{\infty }\exp\left(-\beta BJ_{b}{\;}^{2}\right)dJ_{b}$$ (18)

for various temperatures where *β=hc*/*kT.* This determined an effective *B* according to

$$B_{\text{eff}}=\pi /\left(4\beta {\left(Q\right)}_{B}{\;}^{2}\right).$$ (19)

The corresponding contribution to the Wilson centrifugal effect constant then followed as

$${\left(\rho_{\text{eff}}\right)}_{B}={\left(2T\right)}^{-1}\ln\left(B_{0}/B_{\text{eff}}\right).$$ (20)

A similar procedure was used for the “*A*” rotation. No appreciable contribution came from the “*C*” case.

**Figure 5:**
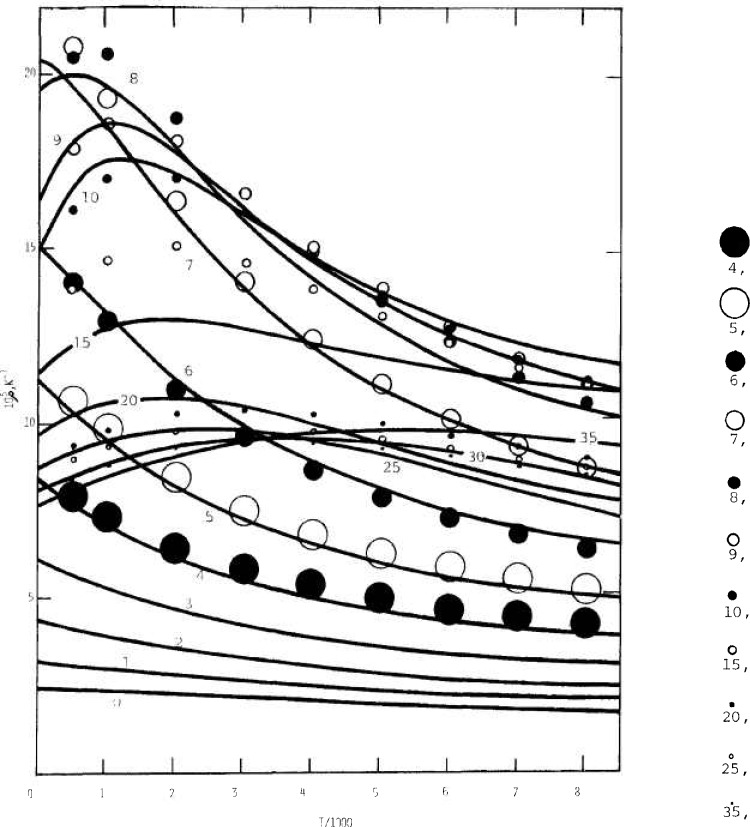
Wilson stretching constant for H_2_O versus temperature and bending vibrational quantum number. Curves are by eq (21), points by quadratures. Values of *v*_2_ for points are

The combined contributions to the Wilson centrifugal effect constant based on the rigid bender model are shown in figure 5 for values of *v*_2_ ranging by unit steps from 4 to 9 and also for 10, 15, 20, 25, and 35. (The sizes of symbols in the figure are not intended to indicate relative importance of the plotted values.) Corresponding curves are shown for the empirical representation

$$10^{5}\rho =\left(b_{0}+b_{1}t+b_{2}t^{2}\right)/\left(1+d_{1}t+d_{2}t^{2}\right),$$ (21)

where *t=T*/1000. For these, the numerical parameters that follow are based on combined estimates as from eq (21), fitted approximately by inspection and graphical processes. With *v* representing *v*_2_, the constants *b_i_*, and *d_i_*, were taken as

$$\begin{array}{l} b_{0}=2.2616\left(1+0.242v+0.037v^{2}+0.00083v^{3}\right)÷\left(1-0.0175v+0.0033v^{2}\right)+8/\left[1+0.8{\left(v-7.3\right)}^{2}\right]\\ b_{1}=1/\left\{.122+{\left[-1.6+1÷\left(0.31+0.02v+0.0019v^{2}-0.000028v^{3}\right)\right]}^{2}\right\}\\ b_{2}=1/\left[1.3+0.106{\left(12-v\right)}^{2}+\left(1E-06\right){\left(12-v\right)}^{6}+\left(2E-08\right){\left(12-v\right)}^{8}\right]-1/\left[5+14.3{\left(8.3-v\right)}^{2}\right]\\ d_{1}=12.6/\left[35.+{\left(v-5\right)}^{2}\right]\\ d_{2}=3./\left[25.+{\left(v-11\right)}^{2}\right] \end{array}$$

as an approximate representation of the calculated values.

**Figure 6:**
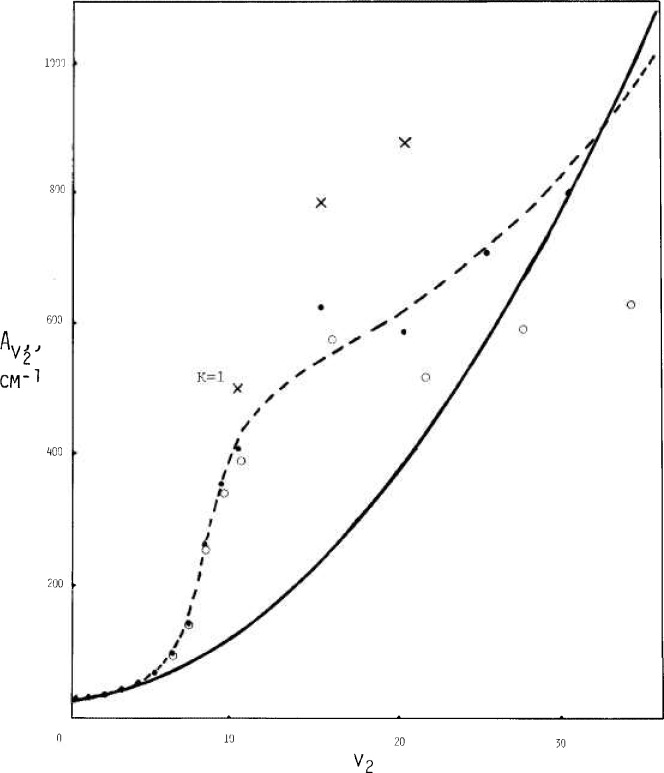
Estimates for the rotational constant *A_v_*_2_ versus *v*_2_ — solid circles via rigid bonds, open circles with bond stretch. The dashed curve is by eq (22), the solid curve via Benedict.

**Figure 7:**
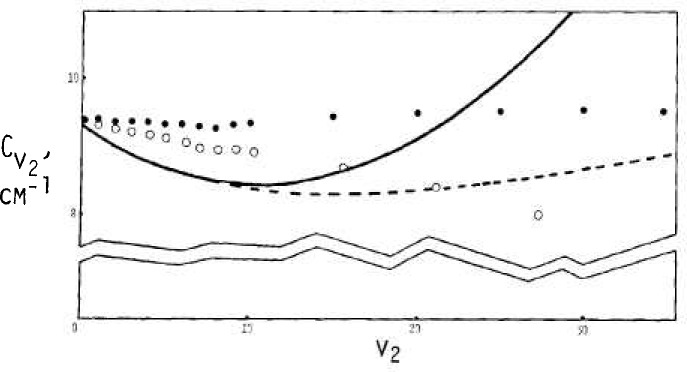
Rotational constant $B_{v_{2}}$ for rotation about intermediate axis - solid circles via rigid bonds, open circles with bond stretch. The dashed curve is by eq (23), the solid curve via Benedict.

**Figure 8:**
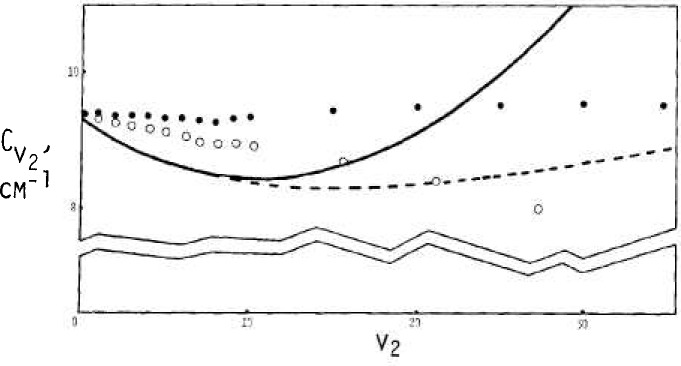
Estimates for the rotational constant $C_{v_{2}}$ versus *v*_2_ solid circles via rigid bonds, open circles with bond stretch. The dashed curve is by eq (24), the solid curve via Benedict.

Values for *A_v_, B_v_*, and C*_v_* as interpreted via data of figures 2, 3, and 4 are shown in figures 6, 7, and 8 by solid circles. The solid curves are from a fit of spectroscopic data by Benedict [8] running up to *v*_2_=4, with *v*_1_ and *v*_3_ also extending up to 4.

The *x*’s in figure 6 show individual estimates at the quantum number *K*=1, indicating that for *A_v_* extrapolation above *v*_2_=7 encounters some imperfection in the traditional representation.

The dashed curves in figures 6, 7, and 8 are given respectively by

$$A=27.8847\left(1+\left\{0.0895v+0.0228v^{2}+\left(0.0022v^{2}+0.00012v^{3}-0.000185v^{4}+0.35E-04v^{5}-0.7E-06v^{6}\right)/\left[\left(1-0.17v+0.008v^{2}\right)\times {\left(1-0.15v+0.007v^{2}\right)}^{2}+1E-06v^{5}\right]\right\}\right)$$ (22)

$$B=14.5118\left\{\left(1.0012771+0.05266v+0.00779v^{2}\right)÷\left(1+0.04v+0.008v^{2}\right)+\left(2.51E-08v^{3}+6.4E-06v^{4}+6.3E-06v^{5}\right)/\left(1+0.04v+0.008v^{2}\right)+0.009\left(v-7\right)/\left[{\left(0.00032\right)}^{2}+{\left(v-7\right)}^{2}\right]\right\}$$ (23)

$$C=\left[9.2806+0.0073v\left(v-1\right)\right]/\left(1+H+H^{2}+H^{3}\right)$$ (24)

with *H*=0.1473*v*/9.2806, where *v* represents *v*_2_.

The expressions for *A_v_* and *B_v_* are roughly representative of the directly indicated model results as shown by the solid circles. For *C_v_*, the equation here is basically a rearrangement of Benedict’s equation, although the model results suggest that a different curve might be better.

The open circles show values that have been adjusted from the solid circles according to an allowance for bond stretching based on OH bond data. The effects may be summarized in part as due to the “effective” *v*_2_ value, v_2_ (eff), being less than the true *v*_2_ in accord with

$$v_{2}\left(\text{eff}\right)=v_{2}/\left[1+\left(1.60E-03\right)v_{2}+\left(6.12E-05\right)v_{2}{\;}^{2}-\left(5.81E-07\right)v_{2}{\;}^{3}+\left(9.7E-09\right)v_{2}{\;}^{3}\right].$$ (25)

This causes the adjusted value for the “true” *v* corresponding to each model estimate to occur for a larger value than the *v* (eff). The change in bond length also reduces the magnitudes of principal rotational constants from the model according to the square of the same ratio.

**Figure 9:**
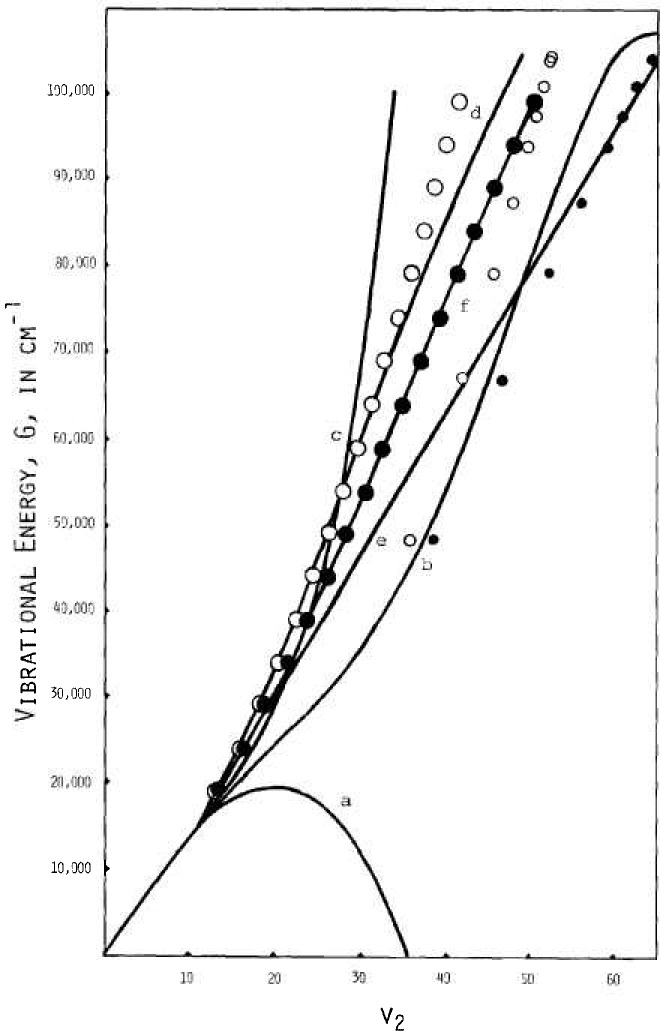
Vibrational bending energy, Small circles: free rotation. Large circles: with bending potential. Open circles: rigid bonds. Solid circles: with bond stretch. Curves: (a) 3 term; (b) with 2 terms added to (a); (c) 4 term fit, eq (26); (d) rational function; (e) for (d) with stretch; (f) combined locus, eq (27).

An indication via the bending model as to the dependence of vibrational energy on the *v*_2_ quantum number is also given directly by the *v*_2_+l/2 values versus *E*_0_ values from the WKBJ integrals with *J_i_*^2^=0. The results are shown graphically in figure 9 by the large open circles, obtained directly using the bending model without any bond stretching allowance. Ad hoc adjustments for bond stretching were then taken from *v*_2_+1/2 differences between the small open circles for free rotation without bond stretch and the small solid circles for free rotation with bond stretch obtained with bond data transferred from the OH bond of the OH diatomic molecule.

As to the curves of figure 9, the one labelled “a” is for three terms in *v*_2_ when *v*_1_=*v*_3_=0, from a fit by Benedict with ∑*v*_1_’s up to 4. Curve “c,” as

$$G=1608.034v_{2}-11.748v_{2}{\;}^{2}-1.643v_{2}{\;}^{3}+0.0937v_{2}{\;}^{4},$$ (26)

is obtained versus *v*_2_ alone from basically the same data with a fourth term included in the fitting. Curve “b,” used in the ideal gas calculations of 1979 [1], was obtained from “a” by adding the two terms

$$0.05v_{2}\left(v_{2}-1\right)\left(v_{2}-2\right)\left(v_{2}-3\right)-0.00051v_{2}\left(v_{2}-1\right)\left(v_{2}-2\right)\left(v_{2}-3\right)\left(v_{2}-4\right).$$

Curve “d” is represented by a rational function with coefficients chosen to fit the large open circles from the potential model without bond stretch. Curve “e” is also by a rational function, but fitted only to the large solid circles at low quantum number. The last curve, “f,” involves a combined locus asymptotic to a rational function curve at low *v*_2_ and to a straight line at large *v*_2_. It is given by

$$G=\left(a+b\right)/2+{\left[{\left(a-b\right)}^{2}+4c^{2}\right]}^{1/2}/2-d$$ (27)

where *a*=2300(*v* −7), *c* = 1600, *d* = 157.4661132, and *b* = 1601.337*v*(1 − 0.4105368*v* + 0.0706926*v*^2^ − 0.006001528*v*^3^ + 0.0002295548*v*^4^) ÷ (1−0.4028641 *v* + 0.06850098*v*^2^ −0.005819278*v*^3^ + 0.0002301345*v*^4^) where *v* represents *v*_2_. It is this curve that represents values used in the present calculation of thermodynamic functions.

In comment on the many digits used for these constants, this is to be attributed not to any extreme accuracy, obviously, but at least in part to a regard for correlation between coefficients and to a desire to retain significance in difference type effects.

It is perhaps well to admit at this point that great accuracy is not claimed for the bending potential used. The application of the bending model is seen as quite successful, however, in providing a clear indication of the rather moderate magnitude of change in distant extrapolation as compared with the results for free rotation. Still, the uncertainty in these extrapolations must be very appreciable.

## Centrifugal Data

As shown in an earlier publication [1], the effects on the rotational partition function for a given vibrational state due to centrifugal distortion and bond stretching may be obtained in semi-classical approximation from the integral

$$Q=8\pi^{2}h^{-3}\int \int_{-\infty }^{\infty }\int \exp\left(-H/kT\right)dp_{x}dp_{y}dp_{z}$$ (28)

with *H=hc*(*W*_0_*+*H_1_), where *W*_0_*=∑B_i_P_i_*^2^ and where *H*_1_ represents the remaining part of the Watson-type rotational Hamiltonian. This uses *B*_1_, = *C_v_*, *B*_2_*=B_v_* and *B*_3_*=A_v_*, the principal rotational constants, with *P*_1_=*P_x_, P*_2_*=P_y_* and *P*_3_=*P_z_.*

The present application of the method has been carried to the evaluation of five coefficients versus temperature. In the 1979 application of the method, the evaluation was made to three coefficients, only. The hope was that the added detail would provide a better overall representation of thermal effects implied by the spectroscopic data.

There may typically be about 20 to 30 terms in current realizations of *H*_1_ involving coefficients of various powers or products of powers of *P*^2^*=P_x_*^2^*+P_y_*^2^*+P_z_*^2^, *“P_xy_^2^”=P_x_*^2^−*P_y_*^2^, and *P_z_*^2^. The factor exp(−*hcH*_1_/*kT*) may be expanded as a Taylor series in powers of *H*_1_. With 20 terms in *H*_1_, running generally up to the 10th power in *P_i_’*s, but with a 12th power in *P_z_*, there are found to be 98 terms in *H*_1_^2^ and 35 in *H*_1_^3^ in the range through the 12th power in *P_i_*. When the terms in *p^2^* and *P_xy_*^2^ are expanded in powers of *P_x_*^2^, *P_y_*^2^ and *P_z_*^2^, the number of separate terms to use in evaluating Gaussian integrals becomes quite large. For each, the integrand is a product over *i*=1, 2, and 3 of *P_i_*^2^*^n^* exp(−*hcB_i_P_i_*^2^/*kT*). As a result, each separate term is of the form

$$Q_{v}=Q_{v}{\;}^{0}\prod_{i=1,2,3}F\left(n_{i}\right){\left(kT/hcB_{i}\right)}^{n_{i}}$$ (29)

where $F(n_{i})=2^{-2n_{i}}(2n_{i})!/n_{i}!$. *Q_v_*^0^ is for a classical rigid rotator for the level (*v*_1_, *v*_2_, *v*_3_), symbolized by “*v*” as indicated earlier. The *F*(*n_i_*) constants are simple fractions that are functions of *n_i_* such as *F*(0)=1, *F*(1) = 1/2, *F*(2)=3/4, *F*(3)=15/8, *F*(4) =105/16, *F*(5)=945/32, *F*(6)=10395/64, etc.

A computer program has been arranged for carrying out the preparation of the correction factor as a series in powers of temperature using the empirical constants of the Watson-type Hamiltonian. With terms up to the 5th power of *H*_1_ covered, the corresponding coefficients are computed to give the logarithm of the correction factor as a series

$$h_{v}=\rho_{0}T\left(1+a_{1}T+a_{2}T^{2}+a_{3}T^{3}+a_{4}T^{4}\right).$$ (30)

In practical application, this has not appeared to provide a well-behaved form when used for moderately high temperatures. Accordingly, the program next computes the corresponding coefficients in a Padé approximant or rational function form,

$$h_{v}=\rho_{0}T\left(1+c_{1}T+c_{2}T^{2}\right)/\left(1+d_{1}T+d_{2}T^{2}\right),$$

or

$$h_{v}=\left(b_{0}T+b_{1}T^{2}+b_{2}T^{3}\right)/\left(1+d_{1}T+d_{2}T^{2}\right).$$ (31)

The coefficients in the Padé form follow from those preceding according to the relations

$$\begin{array}{l} d_{1}=\left(a_{1}a_{4}-a_{2}a_{3}\right)/a_{2}{\;}^{2}-a_{1}a_{3}\\ d_{2}=\left(a_{3}{\;}^{2}-a_{2}a_{4}\right)/a_{2}{\;}^{2}-a_{1}a_{3}\\ c_{1}=a_{1}+d_{1}\\ c_{2}=a_{2}+a_{1}d_{1}+d_{2}. \end{array}$$

The Padé form appears to be much better adapted to computation in ordinary circumstances. For some higher vibrational states, however, there can still be complications such as the occurrence of negative values for the coefficients *c*_2_ and *d*_2_ which are for the highest powers of *T.* Happenings of this type appear to be somewhat dependent on the source of the empirical Hamiltonian constants used.

The program is interactive in asking for values for the principal rotational constants, *A, B*, and *C*, and then for the highest power of temperature to be covered (up to 6 as set up). It then asks progressively, in a selected order for values for 30 coefficients in the Watson Hamiltonian, identifying each by a coefficient name in “string-variable” form, e.g., DELJ, etc. These happen to be in the order shown schematically by:

$$\begin{array}{l} H=-\text{DELJ}*J**4-\text{DELJK}*\text{JZ}**2*J**2\\ \;-\text{DELK}*\text{JZ}**4-2*\text{SDLJ}*\text{JXY}**2\\ \;-\text{SDLK}*(\text{JZ}**2*\text{JXY}**2\\ \;+\text{JXY}**2*\text{JZ}**2)+\text{HJ}*J**6\\ \;+\text{HJK}*\text{JZ}**2**J**4+\text{HKJ}*\text{JZ}**4*\text{J}**2\\ \;+\text{HK}*\text{JZ}**6+2*\text{SHJ}*\text{JXY}**2*J**4\\ \;+\text{SHJK}*(\text{JZ}**2*\text{JXY}**2)\\ \;+\text{JXY}**2*\text{JZ}**2)*J**2\\ \;+\text{SHK}*(\text{JZ}**4*\text{JXY}**2+\text{JXY}**2*\text{JZ}**4)\\ \;+\text{CLJ}*J**8+\text{CLK}*\text{JZ}**8\\ \;+\text{CLKKJ}*\text{JZ}**6*J**2\\ \;+\text{CLJK}*\text{JZ}**4*J**4+\text{CLJJK}*\text{JZ}**2*J**6\\ \;+2*\text{SLJ}*\text{JXY}**2*J**6\\ \;+\text{SLK}*(\text{JZ}**6*\text{JXY}**2+\text{JXY}**2*\text{JZ}**6)\\ \;+\text{SLKJ}*(\text{JZ}**4*\text{JXY}**2\\ \;+\text{JXY}**2*\text{JZ}**4)*J**2\\ \;+\text{SLKJ}*(\text{JZ}**2*\text{JXY}**2\\ \;+\text{JXY}**2*\text{JZ}**2)*J**4+\text{CPK}*\text{JZ}**10\\ \;+\text{CPKKJ}*\text{JZ}**8*J**2\\ \;+\text{CPKJ}*\text{JZ}**6*J**4\\ \;+\text{SPK}*(\text{JZ}**8*\text{JXY}**2+\text{JXY}**2*\text{JZ}**8)\\ \;+Z12*\text{JZ}**12+Z10P2*\text{JZ}**10*J**2\\ \;+Z14*\text{JZ}**14+Z16*\text{JZ}**16\\ \;+Z18*\text{JZ}**18, \end{array}$$ (32)

where J**2 is JX**2+JY**2+JZ**2 and JXY**2 is JX**2−JY**2.

Values for the Padé constants as obtained from the available Watson Hamiltonian constants for the eight observed vibrational levels, based largely on the work of Camy-Peyret and Flaud [7] are given in table 1.

A listing of the program is included in the appendix. Further discussion of the results will be reserved for a later section dealing with table comparisons.

## Rotation at High Temperature

While the Padé form for centrifugal effect seems better adapted for calculation than the simple power series form which encounters convergence problems of an erratically varying sign type, there are other consideration if extrapolation to very high temperature is required.

The rotational quantum numbers can increase up to some limiting large values as “*J_a_*,” “*J_b_*”or “*J_c_*,” beyond which centrifugal force would cause the molecule to break apart by bond rupture. The limiting rotational energy for rotation about any principal axis would be of a general magnitude indicated by

$$D_{R}=B_{i}J_{i}{\;}^{2}$$ (33)

where *B_i_* refers to *A_v_, B_v_* or *C_v_*, according to the axis involved. However, the affected moment of inertia at bond rupture would be appreciably increased by bond stretching over its ordinary value. A semi-classical OH bond model study on the principal rotational constants at zero vibration suggests that the ratio “r” between principal constants at maximum versus at low rotational quantum number should be about 0.195 as r*_A_* for *A*, 0.260 as r*_B_* for *B*, and 0.310 as r*_C_* for *C*.

This leads to the interesting inference that if a limiting partition function would be equal to the “volume” of an ellipsoid with semi-axes *J_a_, J_b_* and *J_c_*, one may estimate the volume as

$$Q_{M}=(4/3)\pi \sigma^{-1}{\left(D_{R}/A_{v}\right)}^{1/2}{\left(D_{R}/B_{v}\right)}^{1/2}\times {\left(D_{R}/C_{v}\right)}^{1/2}R$$ (34)

where *R*, the factor of centrifugal increase, is estimated as

$$\dot{R}={\left(r_{A}r_{B}r_{C}\right)}^{-1/2}=8.0$$ (35)

for low vibrational states. For high vibrational states, where little additional rotational energy is needed to bring about bond breaking, the ratio needed may be much nearer to unity. A form

$$R=[1+(R_{0}-1){\left(D_{v}/D_{0}\right)}^{s}]$$ (36)

with *R*_0_=8.0 and possibly *s*=1 may be a useful speculation as to plausible behavior. Here *D_v_* is the additional energy *D*_0_−*G_v_* to reach dissociation for the vibrational state without rotation.

As to acceptable values for *D_R_* for higher vibrational states, it appears useful, with *D_M_* as dissociation energy including rotation, to note that by a logarithmic plot of *y*=(*D_M_−D*)/*D* versus *x*=(*D−G_v_*)/*D* for the OH diatomic potential, an approximate representation is *y*=(2/9)*x*^7/8^. Estimates of a similar magnitude can also be found from the expression *y*=(1/4)*x*, which is a form easier to use. The latter choice provides an approximate relation *D_R_*=*D*(*x*+*y*), or *D=D−G_v_*+(*D−G_v_*)/4= 1.25(*D−G_v_*).

As in the discussion leading to eq (34), an estimate for the rotational partition function may be based on an integral using an ellipsoidal shell with semi-axes *ħ*(*E*/*A*)^1/2^, *ħ*(*E*/*B*)^1/2^ and *ħ*(*E*/*C*)^1/2^. The “volume” of the shell between energies *hcE* and *hc*(*E+dE*) is 2*π ħ*^3^ (*ABC*)^1/2^ *E*^1/2^ *dE.* In the evaluation of density of states as measured by $\prod_{1}^{3}\left(h^{-1}dp_{i}dq_{i}\right)$, there is a factor 4*π* for orientation of the total momentum vector and 2*π* for position of the rotator in making one revolution. Thus the number of states available within the energy shell needs the factor 8*π*^2^*h*^−3^ to be included, giving

$$dN=2{\left(ABC\right)}^{-1/2}E^{1/2}dE.$$ (37)

An additional factor $f=\left(1+\sum_{i}r_{i}E^{i}\right)$ may represent the increase due to centrifugal effects. Integration to infinite energy gives

$$Q_{R}^{\infty }=\int dQ_{R}=2{\left(ABC\right)}^{-1/2}\int_{0}^{\infty }\exp(-hcE/kT)\times (1+\sum r_{i}E^{i})E^{1/2}dE$$ (38)

or

$$Q_{R}^{\infty }=2{\left(kT/hc\right)}^{3/2}{\left(ABC\right)}^{-1/2}\int_{0}^{\infty }\exp(-x)x^{1/2}\times [1+\sum r_{i}{\left(kT/hc\right)}^{i}x^{i}]dx$$ (39)

The result is

$$\begin{array}{l} Q_{R}^{\infty }=\pi^{1/2}{\left(kT/hc\right)}^{3/2}{\left(ABC\right)}^{-1/2}\\ \;\times [1+(3/2)r_{1}(kT/hc)+(3/2){\left(5/2\right)}_{r_{2}}{\left(kT/hc\right)}^{2}\\ \;+(3/2)(5/2)(7/2)r_{3}{\left(kT/hc\right)}^{3}+\ldots ] \end{array}$$ (40)

Using $r_{i}=\{\prod_{j=1}^{i}\left[2/\left(2j+1\right)\right]\}e_{i}$, this may be identified with eq (41)

$$Q_{R}^{\infty }=\pi^{1/2}{\left(kT/hc\right)}^{3/2}{\left(ABC\right)}^{-1/2}[1+\sum e_{i}{\left(kT/hc\right)}^{i}].$$ (41)

The last factor is *f_v_* or exp(*ρT*) for centrifugal effects according to eq (2).

If the integration is extended only to a rotational energy *E*=*D*, or for *x* to *x*=*hcD*/*kT*, the result for each term involves an incomplete gamma function.

$$Q_{R}^{\infty }=2{\left(kT/hc\right)}^{3/2}{\left(ABC\right)}^{-1/2}[\gamma (3/2,x_{1})+\sum r_{i}\gamma (i+3/2,x_{1}){\left(kT/hc\right)}^{i}].$$ (42)

The recurrence relation *γ*(*a*+1,*x*)=*aγ*(*a,x*)−*x*^a^exp(−*x*) is used to relate all later terms to the first one. Values can be found for *γ*(3/2,*x*)=(*π*^1/2^/2) *H*(*z*)−*z* exp(−*z*^2^), where *x=z*^2^, using *H*(*z*)=1−*π*^−1/2^exp(−*z*^2^)(*z*+0.2)*z*1^4^[1−*z*1^4^(2.5803−2.8136 *z*1+4.0745 *z*1^2^−1.2142 *z*1^3^+1.1657 *z*1^4^−0.0091 *z*1^5^)], where *z*1=1/(*z*+0.1)^1/2^.

The result for a given rotational state may be written as

$$Q_{R}{\;}^{D}=Q^{0}(Q_{x}f_{v}-Q_{s})$$ (43)

with *Q*^0^=*π*^1/2^(*kT/hc*)^3/2^(*A_v_B_v_C_v_*)^−1/2^ *σ*^−1^, as for a rigid rotator, *σ* being the symmetry number, with

$$Q_{x}=2\pi^{-1/2}\gamma (3/2,x_{1})$$

and with *f_v_* representing exp(*ρT*) as in eq (2) or as used for $Q_{R}^{\infty }$. Thus *f_v_* might be used in any form that would appear suitable, such as with a Padé approximant, if acceptable. *Q_s_* is a residual quantity

$$Q_{s}=2\pi^{-1/2}x_{1}{\;}^{1/2}\exp(-x_{1})\sum e_{i}{\left(kT/hc\right)}^{i}\times \left(\sum_{m=1}^{i}\{\prod_{j=1}^{m}\left[2/(2j+1)\right]\}x_{1}{\;}^{m}\right),$$

a form capable of further examination.

As a variant study based on the derivation leading to eq (38), one may remove the Boltzmann factor exp(−*hc E/kT*) and consider the integration up to an energy *E.*

$$Q^{E}=2{\left(A_{v}B_{v}C_{v}\right)}^{-1/2}\int_{0}^{E}(1+\sum e_{i}E^{i})E^{1/2}dE.$$ (44)

This was tried on the ground state and some others. Conversion of the polynomial to a Padé-Wilson exponential form

$$f^{\prime }=\exp[(b0E+b1E^{2}+b2E^{3})÷(1+d1E+d2E^{2})]$$ (45)

quieted a term-wise sign fluctuation effect. However, in extension to very large *E*, a condition of excessively large computed *Q^E^* was encountered. This was due to the exponential factor becoming grossly over-sized. For the ground state, as *E* rises from 1.*E*+5 to 1.*E*+6 cm^−1^, the computed centrifugal factor rises from 6.5 to over 1.*E*+7. This result is contrary to the previous estimate of a limit for the centrifugal factor of the order of *R*=8 or less, as in eq (36). The catastrophe can obviously be avoided by enlarging the denominator by including a term *d*3 *E*^3^, with the ratio *b*2/*d*3 near to 2 or to ln *R.*

Logically, the parameters should be chosen again in such a way that the expansion into a power series would remain unchanged through the first five coefficients. If *s* represents *b*2/*d*3, and with *B*1, *B*2, *D*1 and *D*2 to represent original values of *b*1, *b*2, *d*1 and *d*2, respectively, the revised coefficients can be obtained from

$$\begin{array}{l} s1=b1**3-2*b0*b1*b2+b0**2*b2*d1\\ \;-b0*b1**2*d1+b0**2*b1*d2\\ s2=b0*b1*d1*d2-b1**2*b2-b0*b2*d1**2\\ \;-b1*b2*d1+2*b0*b2*d2-b2**2\\ \;-b0**2*d2**2\\ b2=B2/[1+(s1/s2)/s]\\ d3=b2/s\\ d2=b2*(D2-b1/s)/B2\\ d1=[b2*(D1-b0/s)-d2*B1\\ \;+D2*(B1-b0*D1)]/B2-b0*D2)\\ b1=B1-b0*D1+b0*d1 \end{array}$$

With parameters so modified, the computed centrifugal factor for the ground state at 1.*E*+5 and 1.*E*+6 reciprocal centimeters showed reductions to 2.73 and 6.54, respectively.

The same type of control adjustment should apparently be applicable to the Padé form in terms of temperature in a normal computation. However, in actual application to a multitude of levels, there could seem to be a possibility that the final Padé constants might not always be positive, due to numerical accident. A requirement that *d*3 and *b*2 be positive can be met by using the absolute value of *B*2 for *b*2, with *d*3=*b*2/*s*. The other parameters follow from

$$\begin{array}{l} b0=B0\\ b1=[\left(B1**2-B0*B2\right)*\left(B1-B0*D1\right)\\ \;-B0*B1*\left(B2-b2-B0*D2\right)\\ \;+B0**2*\left(B0*d3-D1*b2\right)]/DEN\\ d1=[\left(B1*D1-B0*D2\right)*\left(B1-B0*D1\right)\\ \;-B1*\left(B2-b2-B0*D2\right)+B0*(B0*d3\\ \;-D1*b2)]/DEN\\ d2=[\left(B1*D2-B2*D1\right)*\left(B1-B0*D1\right)\\ \;+\left(B2-B0*D2\right)*\left(B2-b2-B0*D2\right)\\ \;+\left(B0*D1-B1\right)*\left(B0*d3-D1*b2\right)]/DEN \end{array}$$

with *DEN*= (*B*1**2+*B*0**2*D2−B0*B2−*B*0*B1*D1).

This might preserve only four instead of five coefficients of the series leading to a Padé development and no absolute guarantee is known to exist against occurrence of a zero denominator.

Another simple scheme for keeping the rotational *Q* below the limiting *Q_M_* value for a given vibrational level has been patterned after the familiar relation of a hyperbola to its asymptotes represented as a combination of loci. With *Q_M_* as an excessive rotational partition function without cutoff, an estimate with cut-off included might be

$$Q=\left(1/2\right)\left(Q_{M}+Q_{r}\right)-\left(1/2\right){\left[4qQ_{r}Q_{M}+{\left(Q_{M}-Q_{r}\right)}^{2}\right]}^{1/2}.$$ (46)

The quantity *q* is to be taken in a convenient form showing an acceptable temperature dependence. Results of a graphical study for rotations about principal axes, using bond stretching of the OH molecule, lead to a provisional suggestion that a usable form might be

$$q=q_{1}z\;\exp\left(-z\right)$$ (47)

where *z*=*q*_2_*hcD/kT*, with *q*_1_=0.4 and *q*_2_=1.8. Other representation schemes may reasonably be more suitable, however.

It is conceded that direct rotational cut-off effects are fairly small even for temperatures at the top of the range of the present tabulation. However, an indirect effect in the extrapolation is not quite so negligible. In making evaluations based on the empirical constants of Benedict, it was found that gross differences in behavior between *v*1 and *v*3 dependences were produced with *v*1 or *v*3 large, particularly as *v*2 was increased so as to be more than a small integer. This characteristic is attributed to the effect of the long range of the extrapolation with equations fitted to data at low quantum numbers only. A more uniform behavior has been obtained by a revised procedure for treating the empirical vibrational energy.

## Energies for High Vibrational Levels

In the last several years the method for estimation of high vibrational levels appears to be changing, involving such new developments as are referred to as localized bond excitation and local mode description [10]. Special potential forms can be used for such calculations with constants converted [11] from empirical values found with a conventional valence bond system formulation and normal coordinate analysis [12]. Potential improvement based on direct comparison between computed and “observed” levels could be an ultimate objective. A hazard at the outset in this approach may be a sensitivity to the correctness of identification or assignment of spectroscopic data on which at least the original numerical constants are based. As to direct *a priori* quantum mechanical calculation of levels for the molecule as a collection of nuclei and electrons, based on general physical constants, it appears that significant advances have been made on this intrinsically difficult endeavor. Somewhat approximate agreement with vibrational fundamentals has been obtained [13,14] but whether a similar quality of prediction could be achieved for higher vibrational levels may be in an area of pure speculation. *A priori* calculation appears informative in regard to excited electronic states [15], in an energy domain beyond the range of the present treatment.

Even if a local mode description will prove ultimately more reliable than the conventional approach, it has appeared expedient to continue for the time being with the older formulation, for which the necessary parameters are at hand. It appears plausible that newly and correctly calculated levels should on the average agree tolerably well with the old values of corresponding description. This is thought to be the usual situation for a group of “interacting levels” in a so-called resonance situation.

The vibrational constants used here are based on a formulation by the late Prof. W. S. Benedict [8], described by him as preliminary. His result can be shown as

$$\begin{array}{l} G\left(v1,v2,v3\right)=3692.5965\;v1+1609.1113\;v2\\ \;+3803.6304\;v3-41.5442\;V1\;V1\\ \;-13.4642\;V2\;V2-48.0343\;V3\;V3\\ \;-28.6309\;V1\;V2\\ \;-164.2450\;V1\;V3\\ \;-19.2960\;V2\;V3\\ \;+0.0927\;V1\;V1\;V1\\ \;-0.9003\;V2\;V2\;V2\\ \;+0.2690\;V3\;V3\;V3\\ \;-0.7760\;V1\;V2\;V3\\ \;+1.9316\;V1\;V1\;V2\\ \;+0.2325\;V1\;V1\;V3\\ \;+1.0522\;V1\;V2\;V2\\ \;+1.1192\;V2\;V2\;V3\\ \;+1.5269\;V1\;V3\;V3\\ \;-0.9318\;V2\;V3\;V3, \end{array}$$ (48)

for levels with resonance shifts removed as indicated earlier.

The present proposed innovation in regard to vibrational energy is to suppress the long range effects of Benedict’s fitting on the basis that the fine details of fit while relevant in the region of fit in the low quantum number range (*vi* < 5) may still not be numerically reliable when extrapolated to large *vi.* For the various “small” quadratic and cubic terms, involving “v products” (=*p*), an extrapolation by replacement of “*p*” by *p*/[1+(*p*/*a*)*^k^*] has been used with *k*=6 and with the parameter “*a*” chosen differently for terms quadratic and cubic in the *v*’s. (65 versus 460) This causes these terms to become small in the approach to the dissociation region.

For the estimation of vibrational levels in the region of large *v*1 and *v*3, the procedure adopted was to take the energy as given primarily by a quadratic jointly in *v*1 and *v*3, much as in the case with a Morse potential in a diatomic molecule. Thus, in the case with *v*2=0, the form for this main part of the vibrational energy becomes

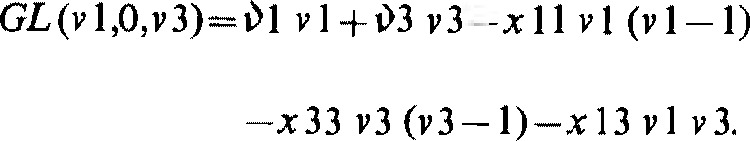
(49)

The anharmonicities for this were chosen so as to agree with energies of dissociative reactions based on thermochemical data. For *v*1 or *v*3 increasing singly with the other at zero, there is dissociation according to H_2_O=O+2H at about 76721 cm^−1^=*D*. For *v*1 and *v*3 equal and advancing together, dissociation is taken to be according to H_2_O=H+OH at about 41280 cm^−1^=*Dm.*

In the cases of *v*1 and *v*3 advancing singly, the energy is given as in

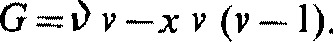
(50)

With 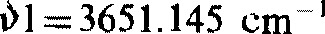 from *G*(1,0,0)−*G*(0,0,0) and also with 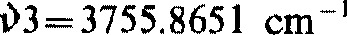 from *G*(0,0,1)−*G*(0,0,0), the corresponding “anharmonicity” constants follow from Birge-Sponer type relations as

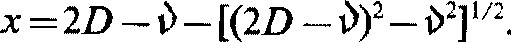
(51)

For *D*=76721 cm^−1^, the long range estimates are *x*11=44.5045 and *x*33=47.1274 cm^−1^. These are raised by about 0.0004 cm^−1^ in covering small residual effects from the suppressed higher order constants at dissociation, which appears to be where *v*1 or *v*3 singly reach a value of about 41.

A somewhat similar procedure using *Dm*=41280 cm^−1^ to estimate *x*13 for *v*1=*v*3=*v* implies the relation

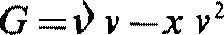
(52)where 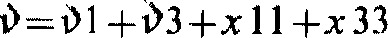 and *x=x*11+*x*33*+x*13. The usual Birge-Sponer relations by

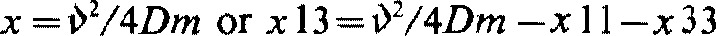
(53)give *x*13=248.95779 kaysers but the partially suppressed residual contributions of other constants at this dissociation energy (near *v*1=*v*3=11) raise *x*13 to 251.3489 cm^−1^.

A multiplier factor [1−0.0028 *v*2−0.00013 *v*2 (*v*2−1)] has been introduced for 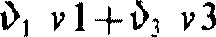 to allow for a diminishing energy increment to dissociation as *v*2 advances upward above *v*2=0. All such adjustments are compensated for in the low quantum number range so as to preserve the behavior there according to the empirical data fit of Benedict [8].

## Thermodynamic Tables for H_2_O

Two sets of tabular values have been included as prospective thermodynamic quantities for the ideal gas state of the light isotopic water molecule. These are here designated by their dates of computation, which were 1982 and 1984.

For the 1982 table, appearing here as table 2, parameter values used were influenced by results of computations for a rigid bender model, adjusted further for bond length increase by centrifugal stretching due to a rotational character of motion in the bending vibration. These included indications as to the *v*2 dependence of the principal rotational constants, the extrapolation of vibrational energy to high *v*2 values, and the course of the ordinary centrifugal effects to high *v*2 and elevated temperatures, using a five parameter Padé formulation.

The 1984 table, shown here as table 3, includes the innovations of the 1982 table, and a few others, also. In the approach to dissociation at high *v*1 and *v*3, the behavior of *Gv* was taken as essentially quadratic in *v*1and *v*3, in resemblance to the known diatomic behavior with a Morse potential. Special functions were used to fade out the detailed higher order terms arising out of Benedict’s *Gv* fit at low vibrational quantum numbers. For eight low-lying vibrational states, numerical values were inserted via the computer program for observed values for vibrational energy, principal rotational constants, and their five member Padé centrifugal parameters, based on reported spectroscopic data analyses using the Watson Hamiltonian formulation. A rotational cut-off approximation of a “locus-asymptote” type was also introduced, but with little apparent effect up to 10000 K.

It is natural to see the difference in values between the two tables as relevant to their uncertainty. It is presumed that the disagreement in values should be attributed to effects in changes in level distribution, which may reflect the ad hoc modification of level description for the later table.

It had appeared reasonable to maintain a favorable view of progress in raising the number of constants based on the Watson Hamiltonian data from three to five. However, it is now recognized in retrospect that some basis for reserve exists. As used, the program for finding Padé constants was able to produce the five parameters as desired even when the Hamiltonian parameters were not complete to a corresponding extent. This might be termed a “spill-over” effect akin to the forming of product terms in a series development. The highest power of *T* directly included as a contribution to “*T* times the Wilson constant” may be obtained by taking the highest net power of *J*’*s* in the Hamiltonian, dividing by 2, and subtracting 1. On this basis, the ground state and first excited vibrational state, (000) and (010), may be “complete” through the 5th power. The states (020) and (030) show fitting in the 4th power, and the states (100), (001), (110) and (o11) include only into the 3rd power. One may hope that a moving of the Padé process into the Hamiltonian will lead to a more uniform treatment [16].

As comment on our present use of a “preliminary” 1972 data formulation received from Professor Benedict [8], we accepted his view that his was better than that of Khachkuruzov [17], of 1959. We note that a more recent vibrational energy formulation presumably of comparable quality was published in 1983 by Bykov, Makushkin and Ulenikov [18], and could in all probability provide a similar basis for a table of thermodynamic quantities.

It appears that greater consideration should be given to recent work such as that by Child and Lawton [19] on local mode representations of vibrational states. However, at this time it is not clear how energies for the entire manifold of vibrational states would be reliably and conveniently given for the calculation of thermodynamic functions on such a basis.

## Conclusion

The objective in this study has been to obtain an improved extrapolation of the ideal gas table to higher temperatures. The procedure has made use of direct data, augmented with numerical estimates based on simple physical models. It is hoped that this might provide a realistic approach to better sum of state estimation.

Although the models have involved some numerical choices that were not at all rigorous, the results may allow such comparisons as may lead to an informed appreciation of the problems remaining for the reduction of uncertainties.

## Figures and Tables

**Table 1:** Constants for low-lying vibrational states from Watson-type Hamiltonian data.

(*v*)	*Gv*	*Av*	*Bv*	*Cv*	*b*0	*b*1	*b*2	*d*1	*d*2
000	0000.0000	27.8806	14.5216	9.2777	2.4518	3.5538	1.0949	1.5266	0.5377
010	1594.7450	31.1284	14.6875	9.1291	3.2111	2.1258	1.0101	0.7945	0.3886
020	3151.6301	35.5867	14.8415	8.9745	4.3691	−.7474	3.1192	.01296	0.7349
100	3657.0532	27.1222	14.3048	9.1046	2.4668	−1.0581	0.6651	−.3572	0.2392
001	3755.9296	26.6480	14.4313	9.1382	2.4097	−.8892	0.5480	−.3130	0.2144
030	4675.1750	42.1323	14.9714	8.8350	6.8534	−4.9258	10.668	−.3500	1.5033
110	5226.5870	30.1712	14.4139	8.9520	3.1892	−1.0880	1.8797	−.2125	0.5829
011	5331.2798	29.5226	14.6136	8.9931	3.0160	−.6923	1.1666	−.1653	0.4035

This includes Padé type Wilson centrifugal effect parameters *B*(*I*) and *D*(*I*) for exp(*ρT*), where *ρ*={*B*(0)+T*[*B*(1)+T*B(2)]}/{1+T*[*D*(1)+T*D(2)]}, with *B*(0)=*b*0/1.0*E*+5, *B*(1)=*b*1/1.0*E*+8, *B*(2)=*b*2/1.0*E*+11, *D*(1)= *d*1/1.0*E*+3, *D*(2)=*d*2/1.0*E+*6

**Table 2:** Thermodynamic quantities for light isotopic water (1982 version).

*T/K*	$\frac{C_{p}^{0}}{R}$	$\frac{S^{0}}{R}$	$-\frac{G^{0}-H_{0}^{0}}{RT}$	$\frac{H^{0}-H_{0}^{0}}{RT}$	$\frac{Q\text{cut}}{Q}$	#
200	4.01111	21.09225	17.11003	3.98222	0.	3
300	4.04064	22.72269	18.72717	3.99552	0.	6
400	4.12079	23.89493	19.87912	4.01581	0.	11
500	4.23672	24.82647	20.77846	4.04801	0.	17
600	4.36880	25.61043	21.52011	4.09032	0.	26
700	4.50951	26.29436	22.15429	4.14007	0.	37
800	4.65679	26.90607	22.71068	4.19539	0.	53
900	4.80868	27.46330	23.20823	4.25507	0.	72
1000	4.96224	27.97789	23.65978	4.31810	0.	95
2000	6.17104	31.83102	26.85729	4.97373	0.	635
3000	6.78260	34.46206	28.97640	5.48566	0.	2366
4000	7.13354	36.46511	30.60809	5.85702	0.	4519
5000	7.38773	38.08513	31.94658	6.13856	0.	6524
6000	7.60934	39.45206	33.08648	6.36559	0.	8080
7000	7.79162	40.63945	34.08250	6.55695	0.	9487
8000	7.90299	41.68807	34.96896	6.71911	0.	10818
9000	7.92822	42.62120	35.76836	6.85284	0.	12075
10000	7.87725	43.45446	36.49608	6.95838	0.	13255

The final column gives the number of vibrational levels involved in the state sum.

**Table 3:** Thermodynamic quantities for light isotopic water (1984 version).

*T/K*	$\frac{C_{p}^{0}}{R}$	$\frac{S^{0}}{R}$	$-\frac{G^{0}-H_{0}^{0}}{RT}$	$\frac{H^{0}-H_{0}^{0}}{RT}$	$\frac{Q\text{cut}}{Q}$	*#*
200	4.01111	21.09218	17.10996	3.98222	0.	3
300	4.04065	22.72262	18.72710	3.99552	0.	6
400	4.12080	23.89486	19.87905	4.01582	0.	11
500	4.23676	24.82641	20.77839	4.04802	0.	17
600	4.36895	25.61038	21.52004	4.09034	0.	26
700	4.50993	26.29435	22.15423	4.14012	0.	37
800	4.65779	26.90615	22.71063	4.19552	0.	53
900	4.81075	27.46355	23.20820	4.25535	0.	72
1000	4.96610	27.97845	23.65980	4.31865	0.	95
2000	6.22473	31.84805	26.86066	4.98740	0.	651
3000	6.83435	34.50332	28.98865	5.51467	3.290E-17	2369
4000	7.13573	36.51478	30.62900	5.88579	3.328E-11	4774
5000	7.34143	38.12993	31.97291	6.15702	2.054E-08	7063
6000	7.52368	39.48479	33.11495	6.36984	4.151E-07	8973
7000	7.67781	40.65664	34.11050	6.54614	9.326E-06	10678
8000	7.77806	41.68916	34.99460	6.69456	3.320E-05	11899
9000	7.80016	42.60601	35.79032	6.81569	2.384E-04	12183
10000	7.76665	43.42708	36.51385	6.91322	4.060E-04	12183

The final column gives the number of vibrational levels involved in the final state sum. The next to the last column gives the fractional reduction in the state sum due to rotational cut-off, according to the “locus-asymptote” estimate used.

## Footnotes

1 Figures in brackets indicate literature references.

## APPENDIX

A listing of the interactive computer program, in BASIC, for carrying out the preparation of the correction factor as a series in powers of temperature using the empirical constants of the Watson-type Hamiltonian:
